# Antagonistic Activity of *Trichoderma* spp. Against *Fusarium oxysporum* in Rhizosphere of *Radix pseudostellariae* Triggers the Expression of Host Defense Genes and Improves Its Growth Under Long-Term Monoculture System

**DOI:** 10.3389/fmicb.2021.579920

**Published:** 2021-03-15

**Authors:** Jun Chen, Liuting Zhou, Israr Ud Din, Yasir Arafat, Qian Li, Juanying Wang, Tingting Wu, Linkun Wu, Hongmiao Wu, Xianjin Qin, Ganga Raj Pokhrel, Sheng Lin, Wenxiong Lin

**Affiliations:** ^1^College of Life Sciences, Fujian Agriculture and Forestry University, Fuzhou, China; ^2^Fujian Provincial Key Laboratory of Agroecological Processing and Safety Monitoring, Fujian Agriculture and Forestry University, Fuzhou, China; ^3^Key Laboratory of Crop Genetic Breeding and Comprehensive Utilization, Ministry of Education, Fujian Agriculture and Forestry University, Fuzhou, China; ^4^Institute of Biotechnology and Genetic Engineering, The University of Agriculture, Peshawar, Pakistan; ^5^Department of Wildlife and Ecology, Faculty of Life Sciences, University of Okara, Okara, Pakistan; ^6^Fujian Provincial Academy of Environmental Science, Fujian Provincial Technology Center of Emission Storage and Management, Fujian, China; ^7^Department of Chemistry, Tribhuvan University, Kirtipur, Nepal

**Keywords:** defense genes, *Trichoderma*, PCR-DGGE, monoculture, *Radix pseudostellariae*

## Abstract

Under consecutive monoculture, the abundance of pathogenic fungi, such as *Fusarium oxysporum* in the rhizosphere of *Radix pseudostellariae*, negatively affects the yield and quality of the plant. Therefore, it is pertinent to explore the role of antagonistic fungi for the management of fungal pathogens such as *F. oxysporum.* Our PCR-denatured gradient gel electrophoresis (DGGE) results revealed that the diversity of *Trichoderma* spp. was significantly declined due to extended monoculture. Similarly, quantitative PCR analysis showed a decline in *Trichoderma* spp., whereas a significant increase was observed in *F. oxysporum*. Furthermore, seven *Trichoderma* isolates from the *R. pseudostellariae* rhizosphere were identified and evaluated *in vitro* for their potentiality to antagonize *F. oxysporum*. The highest and lowest percentage of inhibition (PI) observed among these isolates were 47.91 and 16.67%, respectively. In *in vivo* assays, the *R. pseudostellariae* treated with four *Trichoderma* isolates, having PI > 30%, was used to evaluate the biocontrol efficiency against *F. oxysporum* in which *T. harzianum* ZC51 enhanced the growth of the plant without displaying any disease symptoms. Furthermore, the expression of eight defense-related genes of *R. pseudostellariae* in response to a combination of *F. oxysporum* and *T. harzianum* ZC51 treatment was checked, and most of these defense genes were found to be upregulated. In conclusion, this study reveals that the extended monoculture of *R. pseudostellariae* could alter the *Trichoderma* communities in the plant rhizosphere leading to relatively low level of antagonistic microorganisms. However, *T. harzianum* ZC51 could inhibit the pathogenic *F. oxysporum* and induce the expression of *R. pseudostellariae* defense genes. Hence, *T. harzianum* ZC51 improves the plant resistance and reduces the growth inhibitory effect of consecutive monoculture problem.

## Introduction

Due to the allelopathy and the dysbiosis of microorganisms, continuous planting of many Chinese medicinal herbs in the same land results in a significant decrease in yield and quality, which is known as continuous monoculture problem or soil sickness (Zhang and Lin, 2009; Zhao et al., 2015; Wu et al., 2016). *Radix pseudostellariae* is a perennial herb of the *Caryophllaceae* family, and its tuberous roots are used for medicinal purposes, which has very high economic value (Zhao et al., 2015). However, successive cultivation of *R. pseudostellariae* on the same piece of land leads to a decline in both the quality and yield owing to poor plant performance and insufficient biotic stress resistance (Lin et al., 2015). In consecutive monocultures, previous studies have reported the imbalance in the rhizosphere microbial community of *R. pseudostellariae*, especially the abundance of the pathogenic fungi (*Fusarium oxysporum*) increased significantly under consecutive monoculture (Zhao et al., 2014; Wu et al., 2016a, b, c; Chen et al., 2017). Moreover, most of researches in continuous monoculture problem of *R. pseudostellariae* and their potential biological microorganisms are focused on prokaryotes (i.e., *Pseudomonas* spp. and *Burkholderia* spp.) (Wu et al., 2016a; Chen et al., 2017). Therefore, to develop a reliable system of biological control against plant pathogens, we need to explore the antagonizing role of potentially important eukaryotic microorganisms like fungi as well.

The importance of the beneficial microbes in improving nutrient availability and promoting plant growth, antagonizing soil-borne pathogens, and priming the plant’s immune system is well established and abundantly used in biocontrol strategies (Cotxarrera et al., 2002; Howell, 2006; Lorito et al., 2010; Mendes et al., 2011; Matarese et al., 2012; Walters et al., 2013). *Trichoderma* spp. is a fungal genus in the family *Hypocreaceae*, which is found in the soil, rotting wood, plants, and the ocean. Many species are characterized as opportunistic avirulent, symbiotic and can be used as biological control agents against important plant pathogenic fungi (Harman et al., 2004). For example, *T. harzianum* (SQR-T307) and *T. asperellum* (T-34) are effective biological control agents against *F. oxysporum* (Corrales et al., 2010; Yang et al., 2011). *T. asperellum* isolates could significantly reduce the incidence of tomato wilt when used to suppress *Fusarium* wilt of tomato (Cotxarrera et al., 2002). *T. gamsii* 6085 was used in a competitive test against *F. subtilis* and *F. graminearum*, which confirmed that *T. gamsii* has the ability to antagonize the pathogens of rice (Matarese et al., 2012).

These root-associated mutualistic microbes, besides impacting on plant nutrition and growth, can further boost plant defenses, rendering the entire plant more resistant to pathogens (Romera et al., 2019). To cope with biotic stresses incited by biological agents, like insects and pathogens, plants develop responses, and some of these responses systemically spread far from the infected tissue into the whole plant. These responses include the systemic acquired resistance (SAR) and the induced systemic resistance (ISR) (Shoresh et al., 2010; Pieterse et al., 2014). SAR is induced by insects and pathogens, while ISR is mediated by beneficial microbes present in the rhizosphere, like bacteria and fungi (Mukherjee et al., 2013). Studies have shown that many species of *Trichoderma* could colonize on the root surface that interacts with the first cell layer of the root bark and the epidermis. This symbiotic relationship can effectively protect the plant from pathogens (Mukherjee et al., 2018; Galletti et al., 2020). When *Trichoderma* interacts with plants, it induces the expression of genes involved in the defense responses of plants (Brotman et al., 2013; Mayo et al., 2016; Manganiello et al., 2018; De Palma et al., 2019; Pimentel et al., 2020) and promotes plant growth and root development (Hermosa et al., 2012). However, the role of root-associated mutualistic plant symbiont, *Trichoderma* spp., in activation of *R. pseudostellariae* immunity by triggering the expression of defense-related genes is never explored.

The objectives of this present study are as follows: (1) to analyze the changes of *Trichoderma* communities in rhizosphere soil under *R. pseudostellariae* monoculture using denatured gradient gel electrophoresis (DGGE) combined with quantitative PCR (qPCR) technique and (2) further, to study the effect of different *Trichoderma* strains on the growth and defense response of *R. pseudostellariae* against the *F. oxysporum* and also to assess the expression level of defense-related genes in *R. pseudostellariae* treated with the selected *Trichoderma* isolate.

## Materials and Methods

### Site Overview and Experimental Design

The study was conducted at Ningde City, Fujian Province (27°26′ N, 120°04′ E). This station has a subtropical monsoon climate with an annual mean air temperature of 18.4°C and precipitation of 1,668.3 mm. The root tuber propagation materials of *Radix pseudostellariae* variety “Zhenshen 2” were used as the experimental plant, which was planted on 20th November and harvested on 10th July of the following year. A loam soil was used in the experiments. Physical–chemical characterization of soil used for the experiments was performed, using the protocol described by Jackson (1958) and Watanabe and Olsen (1965). To keep the soil and climatic conditions during the experimental period uniform and subjected to the same field and fertilization management, four types of plots were established within a single experimental field: (1) unplanted soil (CK), (2) containing *R. pseudostellariae* cultivated in fresh soil (FP), (3) plot under cultivation of *R. pseudostellariae* for two consecutive years (SP), and (4) plot under cultivation of *R. pseudostellariae* for three consecutive years (TP). Each type carried three replicate plots with a completely randomized design. The samples were taken in three biological replicates.

### Soil Sampling and DNA Extraction

**FIGURE 1:**
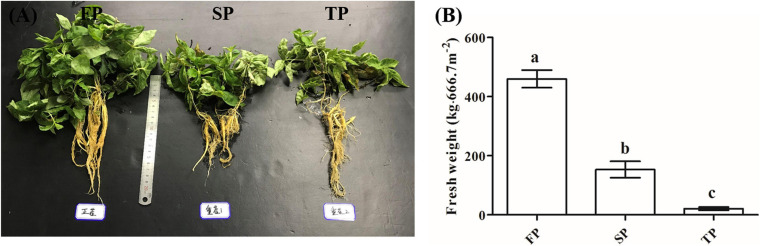
**(A)** Photographs of *R. pseudostellariae* under FP, SP, and TP treatments. **(B)** Yield of *R. pseudostellariae* under FP, SP, and TP treatments. FP, plot with *R. pseudostellariae* cultivated in fresh soil; SP, plot with *R. pseudostellariae*, monocultured for two consecutive years; TP, plot with cultivation of *R. pseudostellariae* for three consecutive years. Different letters show significant differences according to least significant difference (LSD) (*P* ≤ 0.05), *n* = 3.

According to our previous study (Chen et al., 2017), after 5 months of planting *R. pseudostellariae*, its above-ground and underground biomass was significantly different (expanding period of root tubers). Therefore, we randomly collected soil samples from five different locations within each plot on 25th April, 2018 (Figure 1A). Moreover, for yield determination, we harvested the plants on 10th July, 2018 (Figure 1B). While taking soil samples, the rhizosphere soil clung to the root system of *R. pseudostellariae* was collected. DNA of soil (0.5 g) was extracted with BioFast Soil Genomic DNA Extraction Kit (BioFlux, Hangzhou, China) following the instructions. Furthermore, DNA concentration was measured using NanoDrop 2000C Spectrophotometer (Thermo Scientific, United States) and diluted to 20 ngμl^–1^.

### PCR-DGGE Analysis

To evaluate the changes of *Trichoderma* community in the rhizosphere, the nested PCR strategy was designed and applied. In the first round of PCR, we used ITS1F and ITS4, taxon-selective ITS primers. PCR amplification protocol is described in Supplementary Table 1. The 50-μl PCR reaction contains 1 μl of each primer (10 mM), 2 μl template DNA (20 ngμl^–1^), and 25 μl 2X EasyTaq PCR SuperMix (TransGen Biotech, Beijing, China). The products of PCR were subsequently diluted (1:5) for the second PCR reaction via DG-GC (a 40-bp GC-clamp at its 5′ end) and DT primers (Supplementary Table 1). PCR reaction followed for the second round was similar to the first round.

We performed DGGE using the Junyi JY-TD331A system (JUNYI, Beijing, China) using an 8% (*w*/*v*) polyacrylamide gel with a denaturation gradient of 30–60%. The gels were electrophoresed in 1X TAE buffer at 80 V and 60°C for 12 h. After electrophoresis, gels were immediately stained with silver stain.

### Quantitative PCR for *F. oxysporum* and *Trichoderma* Spp.

We performed real-time PCR quantifications of *F. oxysporum* (ITS1F and AFP308R) and *Trichoderma* spp. (DG and DT, as mentioned above) in four soil samples (CK, FP, SP, and TP), and amplification protocol is described in Supplementary Table 1. PCR reaction of 15 μl contains 7.5 μl 2X TransStart Green qPCR SuperMix (TransGen Biotech, Beijing, China), 1 μl of each primer (10 μM), 2 μl of template DNA (20 ngμl^–1^), and 3.5 μl H_2_O. Meanwhile, serial dilutions of plasmid DNA were set as standard curves. The standard curve was generated by log10 value against the threshold cycle (Ct) value. Four independent quantitative PCR assays were performed for each treatment.

### Isolation of *Trichoderma* spp. With Antagonistic Activity Toward *F. oxysporum*

To isolate *Trichoderma* spp., soil suspensions were made by adding 10 g of fresh soil into a flask containing 90 ml of sterile water (10^–1^ gl^–1^). After dilution (10^–2^ gl^–1^), a total of 100 μl suspensions were plated onto Thayer-Martin agar medium (containing 0.25 gl^–1^ pentachloronitrobenzene and 30 mgl^–1^ streptomycin sulfate). The plates were placed in an incubator at 28°C for 4 days and then each single colony was separated and purified.

After purification, we used the CTAB-based method (Rogers and Bendich, 1985) for DNA extraction from different isolates. For sequencing, three primer sets (ITS1F and ITS4, EF1 and EF2, and fRPB2-5f and fRPB2-7cr) were used for amplification (protocol as described in Supplementary Table 1). PCR products were cloned into the pEASY-T1 Cloning vector and sent to BoShang (Fuzhou, China) for sequencing. We further used the BlastN search method to screen for similar sequences in the NCBI and Tricho-BLAST at the website of the International Subcommission on *Trichoderma* and *Hypocrea* Taxonomy. For phylogram, published ITS1, *rpb*2, and *tef*1 sequences for biocontrol isolates were obtained from GenBank. ClustalX2 programs were used for sequence alignment. Gaps/missing data treatment was set to complete deletion. Phylogenetic analysis was carried out with the MEGA6 software. Maximum likelihood was used for statistical method. Neighbor-joining (NJ) trees were constructed for each data set (ITS1, *rpb*2, and *tef*1) using the Tamura–Nei distance measure. The robustness of the internal branches was assessed with 1,000 bootstrap replications (Saitou and Nei, 1987).

### Evaluation of the Biocontrol Effects of *Trichoderma* spp.

For *in vitro* antagonism assays, *Trichoderma* isolates were used to evaluate the biocontrol effects against *F. oxysporum* (pathogenic fungi of *R. pseudostellariae*), which was part of the microbial collection of our lab (Chen et al., 2017). The strains (*F. oxysporum* and *Trichoderma* isolates) were inoculated in potato dextrose agar (PDA) slant culture medium at 4°C. We inoculated each *Trichoderma* isolates in dual culture with *F. oxysporum*. Two different isolates were placed 5.5 cm apart on the same PDA plate with three replicates. After incubation at 30°C for 5 days, the parameters of the antagonistic activity of *Trichoderma* isolates against *F. oxysporum* were recorded. Thus, the percentage of inhibition (PI) was calculated by the following formula:%PI = [(r1 - r2)/r1] × 100, where r1 is the distance between the furthest point and sowing point of the *F. oxysporum* and r2 represents the distance between the sowing point and the edge of the *F. oxysporum* from where *F. oxysporum* and *Trichoderma* mycelia came into contact (Supplementary Figure 1).

Unplanted soil (CK) was used for pot assays. *R. pseudostellariae* were planted in plastic pots and placed in a greenhouse on December 15, 2017. After 5 months of planting, a 2-ml spore suspension (a spore suspension of *F. oxysporum* with a concentration of 10^6^ spores · ml^–1^ was made by rinsing mycelia with sterile water) of isolated *F. oxysporum* was added to the soil for observing the effects of *Fusarium* wilt on *R. pseudostellariae*. In addition, 7 days after inoculation with *F. oxysporum*, spore suspensions of four isolated strains (ZC4, ZC5, ZC51, and CC2-7) were added to pots to evaluate the biocontrol potential of *Trichoderma* spp. Each treatment was three replicates. After 16 days, rhizospheric soil was collected from each treatment (FOX, ZC4, ZC5, ZC51, and CC2-7), and then, soil DNA was immediately extracted for qPCR assays (as mentioned above) of *F. oxysporum* and *Trichoderma* spp.

### Expression Analysis of Defense-Related Genes in *R. pseudostellariae*

To further determine the effect of *Trichoderma* treatment and/or *F. oxysporum* infection on the expression of defense-related genes in *R. pseudostellariae*, we prepared MS medium for *in vitro* culture of *R. pseudostellariae*. Seedlings of *R. pseudostellariae* were transferred in the medium with tweezer and placed in a culture room at 26°C. After 60 days of incubation in the culture room, four treatments were set up: (1) inoculated with *F. oxysporum* into tissue-cultured seedlings of *R. pseudostellariae* (F); (2) inoculated with *T. harzianum* ZC51 into tissue-cultured seedlings of *R. pseudostellariae* (T); (3) simultaneously inoculated with *F. oxysporum* and *T. harzianum* ZC51 into the tissue-cultured seedlings of *R. pseudostellariae* (TF); and (4) tissue-cultured seedlings of *R. pseudostellariae* without any treatment (NTF). Seven days after the inoculation, the tissue culture seedlings of *R. pseudostellariae* were taken out; the plants were washed with sterile water, quickly treated with liquid nitrogen, and frozen in a refrigerator at -80°C for later extraction of RNA.

### Plant RNA Isolation and Real-Time PCR Analysis

Plants were ground into powder with liquid nitrogen, and plant RNA was extracted with TransZol Up Plus RNA Kit (TransGen Biotech, Beijing, China) in accordance with the instructions. Furthermore, RNA concentration was measured using NanoDrop 2000C Spectrophotometer (Thermo Scientific, United States). According to the kit’s instructions, the first-strand cDNA was synthesized using TransScript^®^, miRNA First-Strand cDNA Synthesis SuperMix (TransGen Biotech, Beijing, China). Each sample used 1 μg of total RNA, and the products were immediately diluted to 80 μl with DEPC water as a template.

Based on the previous transcriptome data of *R. pseudostellariae* in our laboratory (Qin et al., 2017), nine primer pairs were used (Supplementary Table 2) to analyze the expression of defense-related genes in *R. pseudostellariae* as a result of *Trichoderma* and/or *F. oxysporum* infection. The actin gene (Supplementary Table 2) was used as an internal reference gene. The 15 μl of the PCR reaction contains 7.5 μl of 2 × SYBR Green qPCR Master Mix (TransGen Biotech, Beijing, China), 1 μl of each primer (10 μM), 0.6 μl of cDNA template, and 5.9 μl H_2_O. The PCR program was as follows: 94°C for 30 s, followed by 40 cycles of 94 C for 5 s and 60°C for 30 s. After RT-PCR, the 2^–Δ^ ^Δ^ ^*CT*^ method (Livak and Schmittgen, 2001) was used to calculate the relative gene expression levels.

### Statistical Analysis

Grayscale of DGGE bands was performed with the Quantity One v4.6.2 software to detect the band of gel. Principal component analysis (PCA) of DGGE was performed by SPSS 20.0 software. Diversity analysis of DGGE was performed by DPS 7.05. For RT-PCR, comparison between two groups was done with independent sample *T*-test by Excel 2013 software. Multiple comparison was carried out by one-way analysis of variance (ANOVA) followed by least significant difference (LSD) test (*P* ≤ 0.05) using DPS 7.05 software for all parameters.

## Results

### The Yield of *R. pseudostellariae* Under Consecutive Monoculture

The yield of *R. pseudostellariae* in the FP was significantly higher (*P* < 0.05) than SP and TP (Figure 1B). The fresh weights of roots in FP were 459.8 kg⋅per 666.7 m^2^, while it was 153.4 kg and 20.9 kg⋅per 666.7 m^2^ in SP and TP, respectively (Figure 1B).

### Soil Nutritional Status

The chemical composition of the soil evaluated was as follows: total nitrogen 1.65 g⋅kg^–1^, available nitrogen 36.42 mg⋅kg^–1^, total phosphorus 0.51 g⋅kg^–1^, effective phosphorus 100.31 mg⋅kg^–1^, total K 7.66 g⋅kg^–1^, and effective potassium 322.52 mg⋅kg^–1^.

### *Trichoderma*-Specific DGGE

**FIGURE 2:**
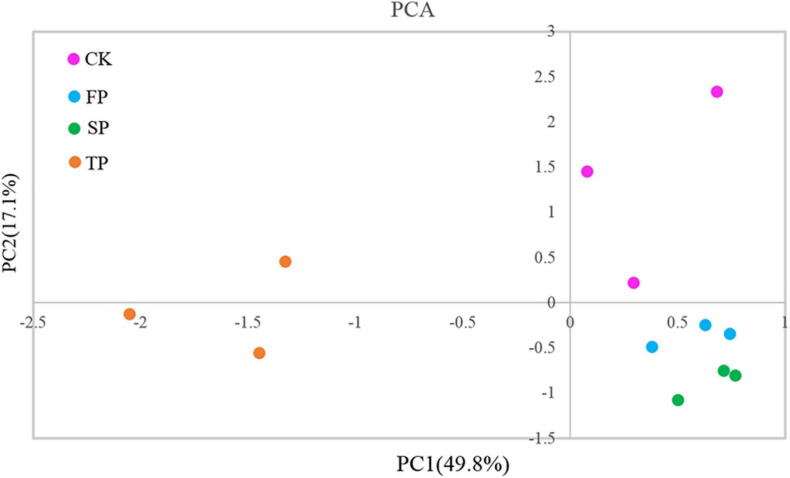
Principal component analysis of *Trichoderma* DGGE. CK, unplanted soil; FP, plot with *R. pseudostellariae* cultivated in fresh soil; SP, plot with *R. pseudostellariae*, monocultured for two consecutive years; TP, plot with cultivation of *R. pseudostellariae* for three consecutive years; DGGE, denatured gradient gel electrophoresis.

*Trichoderma*-specific DGGE analysis indicated that the shifts of the *Trichoderma* community in the rhizosphere changed with increasing period of monoculture (Supplementary Figure 2). Based on DGGE profiles, we performed principal component analysis (PCA) to explore *Trichoderma* rhizosphere community structure between the four different soil conditions. Among them, the first principal component revealed 49.80% of the total variance, and the second principal component indicated 17.10% of the total variance. The results of PCA also showed that the *Trichoderma* community in TP was separated from CK, FP, and SP by the first principal component, and CK was separated from FP and SP by the second principal component (Figure 2).

We also analyzed the diversity of *Trichoderma*-specific DGGE. The results showed that the Simpson, Shannon, and Brillouin’s indices decreased significantly with increasing period of monoculture (*P* ≤ 0.05). However, there was no significant difference in evenness index among the four samples (Table 1).

**TABLE 1:** Estimated Simpson, Shannon, evenness, and Brillouin’s indices for all the samples using *Trichoderma*-specific DGGE.

Treatments	Simpson	Shannon	Evenness	Brillouin
CK	0.9094 + 0.0082a	3.5867 + 0.0571a	0.9693 + 0.0154a	3.5603 + 0.054a
FP	0.9147 + 0.0015a	3.6275 + 0.011a	0.9803 + 0.003a	3.6006 + 0.0094a
SP	0.907 + 0.0016a	3.5073 + 0.0093b	0.9783 + 0.0026a	3.4811 + 0.0084b
TP	0.8835 + 0.0025b	3.1317 + 0.0144c	0.9879 + 0.0046a	3.104 + 0.0136c

*Different letters within a column show significant differences according to LSD (P ≤ 0.05), n = 3. CK, unplanted soil; FP, planting of R. pseudostellariae in the newly planted soil; SP, planting of R. pseudostellariae in two consecutive years; TP, planting of R. pseudostellariae in three consecutive years.*

### Abundance of *Trichoderma* spp. and *F. oxysporum* by Quantitative PCR Under Different Continuous Years

**FIGURE 3:**
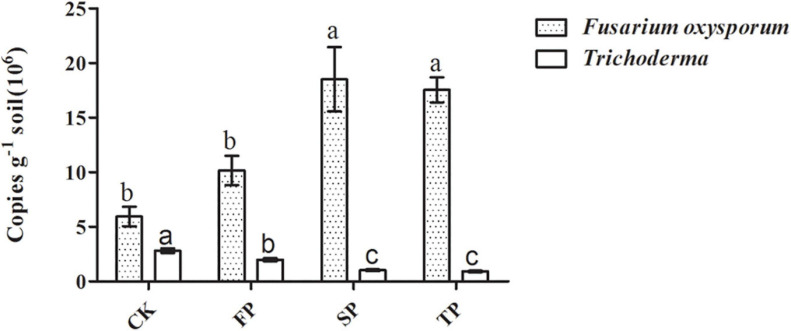
Quantification of *Trichoderma* spp. and *F. oxysporum* in the different plots. CK, control with unplanted soil; FP, plot with *R. pseudostellariae* cultivated in fresh soil; SP, plot with *R. pseudostellariae*, monocultured for two consecutive years; TP, plot with cultivation of *R. pseudostellariae* for three consecutive years. Different letters in the same color show significant differences according to least significant difference (LSD) (*P* ≤ 0.05); data are means ± standard errors (one-way analysis of variance, *n* = 4).

Quantitative PCR was used to analyze changes in *Trichoderma* spp. and *F. oxysporum* abundance in four soil samples (Figure 3). For *Trichoderma* spp. and *F. oxysporum* qPCR analyses, standard curves of *y* = −0.2271*x* + 10.763 (*R*^2^ = 0.9944) and *y* = −0.2673*x* + 10.607 (*R*^2^ = 0.9986), respectively, were developed. Abundance of *Trichoderma* spp. was significantly decreased with prolonged monoculture (Figure 3). These results were consistent with the *Trichoderma*-specific DGGE analysis (Supplementary Figure 2). However, the quantitative PCR results for *F. oxysporum* were the opposite (Figure 3).

### Screening for *Trichoderma* Isolates With Antagonistic Activity Toward *F. oxysporum*

For *in vitro* antagonism assays, we screened seven isolates of *Trichoderma* from four different soils. The results of sequencing showed that the seven isolates belonged to three species of *Trichoderma* (Table 2). The accession number (ITS, *tef*1, and *rpb*2) of seven isolates are in Table 2. Among these, ZC5 isolate showed the highest antagonistic activity (72.77%) (Table 2) toward *F. oxysporum*, whereas ZC13 showed the lowest antagonistic activity.

**TABLE 2:** *In vitro* antifungal activity of *Trichoderma* strains against *F. oxysporum.*

Lab. Code	Inhibition in growth assay	Identify	Accession number (ITS)	Accession number (*rpb*2)	Accession number (*tef*1)
ZC5	47.91 ± 3.41a	*T. harzianum*	MW376900.1	MW407164	MW415424
ZC51	47.66 ± 3.21a	*T. harzianum*	MW376903.1	MW407167	MW415425
ZC4	30.66 ± 1.36b	*T. asperelloides*	MW376899.1	MW407163	WM588808
CC2-7	30.16 ± 11.31b	*T. rugulosum*	MW376897.1	MW407161	MW588806
ESK2	24.41 ± 4.44bc	*T. asperelloides*	MW376898.1	MW407162	MW588807
ZC11	23.62 ± 1.67bc	*T. asperelloides*	MW376901.1	MW407165	MW588809
ZC13	16.67 ± 2.67c	*T. asperelloides*	MW376902.1	MW588811	MW588810

*Different letters within a column show significant differences according to LSD (P ≤ 0.05), n = 3.*

**FIGURE 4:**
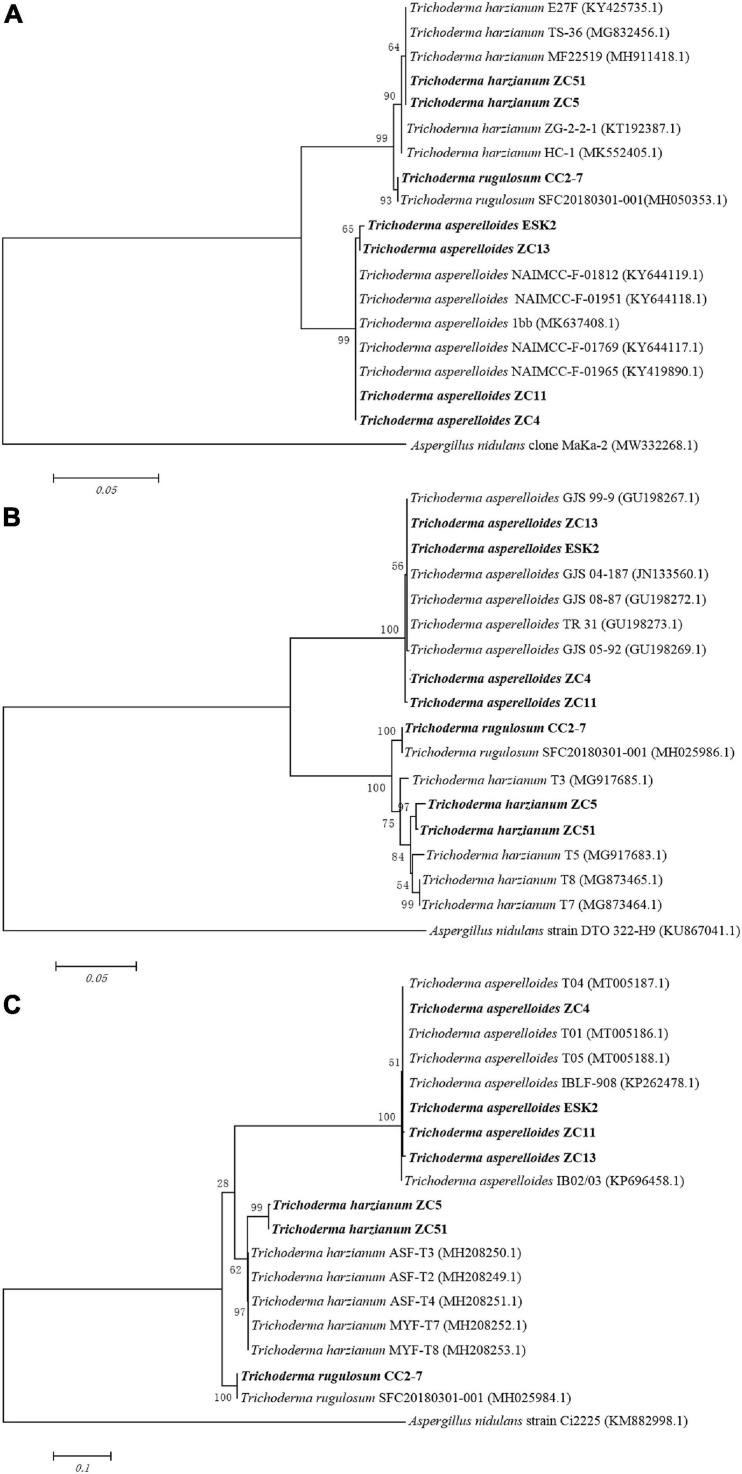
Neighbor-joining tree based on sequence analysis of *Trichoderma* isolates used in this study. Values of the bootstrap analysis (1,000 repetitions) are given at the nodes. **(A)** ITS; **(B)** *rpb*2; **(C)** *tef1. Aspergillus nidulans* was the outgroup. Sequences of biocontrol isolates used for this comparison were obtained from GenBank.

The phylogenetic analysis (ITS) using neighbor-joining method generated a dendrogram with three main branches, where the first branch included *T. harzianum* ZC5 and ZC51; the second branch comprised *T. rugulosum* CC2-7; and the third branch comprised *T. asperelloides* ESK2, ZC13, ZC11, and ZC4 (Figure 4A). For *rpb*2, a dendrogram contained three main branches, where the first branch included *T. asperelloides* ZC13, ESK2, ZC14, and ZC11; the second branch comprised *T. rugulosum* CC2-7; and the third branch comprised *T. harzianum* ZC5 and ZC51 (Figure 4B). For *tef*1, a dendrogram contained three main branches, where the first branch included *T. asperelloides* ZC4, ESK2, ZC11, and ZC13; the second branch comprised *T. harzianum* ZC5 and ZC51; and the third branch comprised *T. rugulosum* CC2-7 (Figure 4C).

### Biocontrol Effects of *Trichoderma* spp.

**FIGURE 5:**
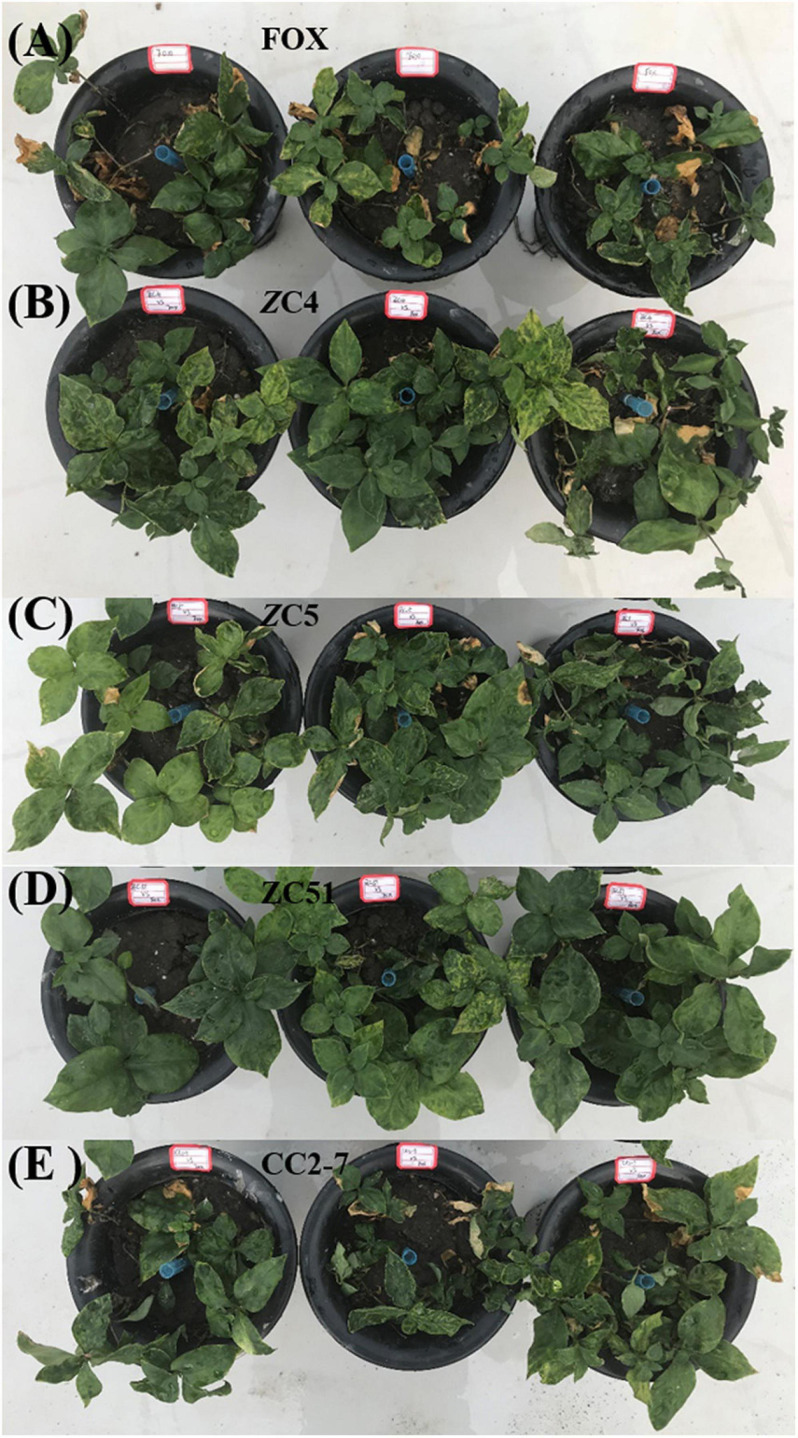
Biocontrol potential of *Trichoderma* against *F. oxysporum*. **(A)** *R. pseudostellariae* was treated with *F. oxysporum* (FOX); **(B)** *R. pseudostellariae* was treated with *F. oxysporum* and *T. asperellum* ZC4 (ZC4); **(C)** *R. pseudostellariae* was treated with *F. oxysporum* and *T. harzianum* ZC5 (ZC5); **(D)** *R. pseudostellariae* was treated with *F. oxysporum* and *T. harzianum* ZC51 (ZC51); **(E)** *R. pseudostellariae* was treated with *F. oxysporum* and *T. hamatum* CC2-7 (CC2-7).

Based on the *in vitro* antagonism assays, four isolates of *Trichoderma* (ZC4, ZC5, ZC51, and CC2-7) that showed inhibition activity against *F. oxysporum* higher than 30% were selected for further *in vivo* biocontrol assay (Figure 5). In the pot experiment, compared with the control (FOX), we found that *T. harzianum* ZC51 significantly inhibited the growth of *F. oxysporum* and enhanced the growth of *R. pseudostellariae*, whereas no disease symptoms developed during the period of the experiment (Figure 5D).

**FIGURE 6:**
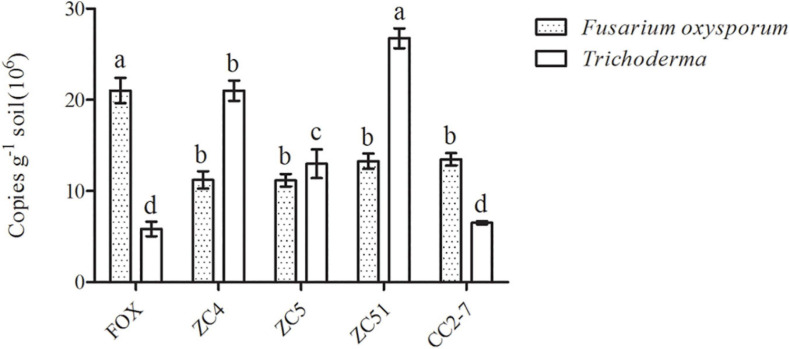
Quantification of *Trichoderma* spp. and *F. oxysporum* in the pot experiment. FOX, *R. pseudostellariae* was treated with *F. oxysporum*; ZC4, *R. pseudostellariae* was treated with *F. oxysporum* and *T. asperellum* ZC4; ZC5, *R. pseudostellariae* was treated with *F. oxysporum* and *T. harzianum* ZC5; ZC51, *R. pseudostellariae* was treated with *F. oxysporum* and *T. harzianum* ZC51; CC2-7, *R. pseudostellariae* was treated with *F. oxysporum* and *T. hamatum* CC2-7. Different letters in the same color show significant differences according to least significant difference (LSD) (*P* ≤ 0.05); data are means ± standard errors (one-way analysis of variance, *n* = 4).

Moreover, quantitative PCR was used to analyze changes in *Trichoderma* spp. and *F. oxysporum* abundance in the pot experiments. Compared with the control (FOX), the abundance of *Trichoderma* spp. in the soil treated with *Trichoderma* strains ZC4, ZC5, and ZC51 increased significantly, with *T. harzianu*m ZC51 showing the highest abundance (Figure 6). Finally, the quantitative PCR results for *F. oxysporum* in the pot experiments showed that all treatments significantly decreased as compared to control (Figure 6). The results showed that *T. harzianum* ZC51 strain, potentially, could be used as a biological control agent against *F. oxysporum.*

### Effect of *Trichoderma* Treatment and/or *F. oxysporum* in the Expression of *R. pseudostellariae* Defense-Related Genes

The *T. harzianum* ZC51 strain was selected, based on its positive effect on *R. pseudostellariae* phenotype without infection (Figure 5D) and the highest abundance among all treatments (Figure 6). The expression of defense-related genes was examined in these plants, i.e., *Trichoderma* ZC51-non-inoculated and *F. oxysporum*-infected plants (F), *Trichoderma* ZC51-inoculated and *F. oxysporum*-non-infected plants (T), *Trichoderma* ZC51-inoculated and *F. oxysporum*-infected (TF) plants, or *Trichoderma* ZC51-non-inoculated and *F. oxysporum*-non-infected plants (NTF).

**FIGURE 7:**
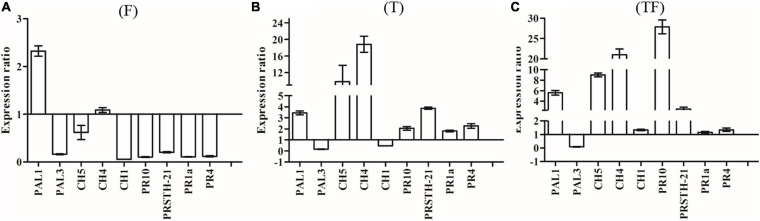
Expression of *PAL1*, *PAL3*, *CH5*, *CH4*, *CH1*, *PR10*, *PRSTH-21*, *PR1a*, and *PR4* genes in comparison with α-actin reference genes. **(A)** Inoculated with *F. oxysporum* into tissue-cultured seedlings of *R. pseudostellariae*; **(B)** inoculated with *T. harzianum* ZC51 into tissue-cultured seedlings of *R. pseudostellariae*; **(C)** simultaneously inoculated with *F. oxysporum* and *T. harzianum* ZC51 into the tissue-cultured seedlings of *R. pseudostellariae*. Data are means ± standard errors (one-way analysis of variance, *n* = 4).

To analyze the expression of defense-related genes, we used actin as a housekeeping gene to determine the relative expression levels of other genes. Expressions of *PAL1* and *PAL3* were studied to determine the involvement of phenylalanine ammonia lyase in *R. pseudostellariae* response to *T. harzianum* ZC51 treatment and/or *F. oxysporum.* There was no significant difference of the *PAL1* and *PAL3* expression in the three treatments (Figures 7A–C). We also examined the expression of *CH1*, *CH4*, and *CH5* of chitinase by treated plants. Expression of *CH4* and *CH5* increased in plants inoculated with *Trichoderma* ZC51 (T) (Figure 7B) and Trichoderma ZC51-inoculated and *F. oxysporum*-infected (TF) (Figure 7C). In contrast, the expression of *CH1* and *CH5* decreased in plants only infected with *F. oxysporum* (F) (Figure 7A). In addition to these genes, we further analyzed the expression of *PRSTH-21*, *PR1a*, *PR4*, and *PR10* involved in the process of plant disease resistance. The expression of *PRSTH-21*, *PR1a*, *PR4*, and *PR10* increased in *Trichoderma* ZC51-inoculated (T) (Figure 7B) and *F. oxysporum*-infected (TF) (Figure 7C) plants. However, the opposite was true for the plants only infected with *F. oxysporum* (F) (Figure 7A).

Also, in order to understand the effect of *Trichoderma* ZC51 (T) on gene expression of *F. oxysporum* (F)-infected *R. pseudostellariae*, we compared the expression of the same gene in different treatments (Table 3). Among these genes, the expression of *PAL1*, *CH5*, *CH4*, *CH1*, *PR10*, *PRSTH-21*, *PR1a*, and *PR4* significantly increased in plants which were treated with T and TF treatments (Table 3).

**TABLE 3:** Expression of *PAL1*, *PAL3*, *CH5*, *CH4*, *CH1*, *PR10*, *PRSTH-21*, *PR1a*, and *PR4* genes in comparison with α-actin reference genes.

Treatments	*F*	*T*	TF
*PAL1*	2.3231 ± 0.1857c	3.4471 ± 0.3011b	5.5851 ± 0.7776a
*PAL3*	0.1622 ± 0.0157a	0.1699 ± 0.0257a	0.0929 ± 0.026b
*CH5*	0.6188 ± 0.2544b	9.8098 ± 6.8382a	8.9884 ± 0.6374a
*CH4*	1.0905 ± 0.0894b	18.8246 ± 3.3248a	21.0104 ± 2.4285a
*CH1*	0.0565 ± 0.0014c	0.471 ± 0.0095b	1.3374 ± 0.1003a
*PR10*	0.1049 ± 0.0136b	2.0501 ± 0.2914b	27.8775 ± 2.9425a
*PRSTH-21*	0.2035 ± 0.0206c	3.872 ± 0.1593a	2.5438 ± 0.5835b
*PR1a*	0.1075 ± 0.005c	1.8026 ± 0.1541a	1.1476 ± 0.1243b
*PR4*	0.1188 ± 0.021c	2.2681 ± 0.3655a	1.3469 ± 0.2162b

*Different letters within a row show significant differences according to LSD (P ≤ 0.05), n = 4. F, inoculated with F. oxysporum into tissue-cultured seedlings of R. pseudostellariae; T, inoculated with T. harzianum ZC51 into tissue-cultured seedlings of R. pseudostellariae; TF, simultaneously inoculated with F. oxysporum and T. harzianum ZC51 into the tissue-cultured seedlings of R. pseudostellariae.*

In general, the results demonstrated that *Trichoderma* ZC51 interaction with *R. pseudostellariae* affected the expression of plant defense-related genes related to the chitinase and pathogenesis-related proteins, but does not involve phenylalanine ammonia lyase.

## Discussion

The low quality and reduced yield of Chinese medicinal herbs are commonly observed due to recurrent cultivation on the same land for many years. This phenomenon of low yield, compromised medicinal quality, poor growth of plants, and high disease susceptibility is owing to consecutive monoculture problems or soil sickness (Zhang and Lin, 2009; Wu et al., 2016c). Our study revealed the facts for typical growth inhibition effects under consecutive monoculture of *R. pseudostellariae*, with poor plant performance and insufficient resistance to disease. Soil physical and chemical properties, accumulation of root exudates, and shift in the soil microbial community are some factors responsible for the consecutive monoculture problem of *R. Pseudostellariae* (Zhang and Lin, 2009; Wu et al., 2016a). The biological relationships between plants and microorganisms in the rhizosphere play a crucial role for the health and growth of a plant, which has been paid much attention in recent days (Haney and Ausubel, 2015; Lebeis et al., 2015).

*Trichoderma* spp. have been studied commonly because of its ability to inhibit soil-borne pathogens and have good plant defense responses (Papavizas, 1985; Hermosa et al., 2012; Pimentel et al., 2020). In this study, PCR-DGGE results showed significant shifts in *Trichoderma* community in the rhizosphere of *R. pseudostellariae* after extended monoculture (Supplementary Figure 2 and Figure 2). Based on PCR-DGGE of *Trichoderma*, results of diversity showed that the extended monoculture of *R. pseudostellariae* significantly decreased the *Trichoderma* spp. diversity (Table 1). Quantitative PCR assay confirmed the decrease in *Trichoderma* with the increasing years of monoculture (Figure 3A), whereas the abundance of *F. oxysporum* was significantly increased (Figure 3B). A previous study has also reported the changes in the composition and diversity of *Fusarium* spp. and increase in the abundance of *F. oxysporum* with the increasing years of monoculture (Chen et al., 2017). This selective change in the microbial community is due to the difference in response of these microorganisms to the root exudates in the rhizosphere (Huang et al., 2014; Zhalnina et al., 2018).

A negative shift in the composition of the soil microbial community is a consequence of the development of soil-borne diseases (Mazzola, 2004). Therefore, maintaining the biodiversity of beneficial soil microbes is crucial to soil health. Biological control with exploitation of the rhizosphere microorganisms that can directly antagonize with plant pathogens is considered to be the most promising method for preventing plant diseases (Qiu et al., 2012; Shen et al., 2014). These species mostly include antagonistic fungi such as *Trichoderma* spp. and *Penicillium* spp. (Cotxarrera et al., 2002; Howell, 2002; Siddiqui and Akhtar, 2009). In this study, most of the isolated strains of *Trichoderma* can inhibit the growth of *F. oxysporum* (Table 2). The difference in antagonistic abilities may be due to genotype variability (Debbi et al., 2018). The *in vivo* assays revealed that *R. pseudostellariae* treated with *T. harzianum* ZC51 has the best growth phenotype without displaying any disease symptom. Yedidia et al. (2001) reported that *T. harzianum* T-203 increased the root length, aerial parts, dry weight, and size of the blade by 75, 95, 80, and 45%, respectively, in cucumber plants. Other studies have shown that *Trichoderma* spp. could promote plant growth, increase nutrient utilization, and improve crop production (Harman et al., 2004).

There is ample evidence that *Trichoderma* species could induce plant defense responses (Yedidia et al., 1999; Gallou et al., 2009; Singh et al., 2011). However, little is known about the effect of *Trichoderma* treatment on the expression of defense-related genes in *R. Pseudostellariae*. PR proteins are well-known proteins that is induced by pathogens and play an important role in the process of plant disease resistance (Linthorst and Van Loon, 1991; Edreva, 2005). Moreover, chitinase proteins a pathogenesis-related proteins that are induced by pathogens; thus, chitinase constitute a crucial part of the plant’s defense against fungal pathogens (Punja and Zhang, 1993; El Hadrami et al., 2010).

As described, the interaction of *R. pseudostellariae* with *F. oxysporum* caused the repression of the seven defense-related genes (*PAL3, CH5, CH1, PR10, PRSTH-21, PR1a*, and *PR4*), via a mechanism to overcome plant defense responses and thereby promoting the process of infection in plants (Peix et al., 2001). Zhao et al. (2003) also reported similar results in experiments with tomato plants infected with *Pseudomonas syringae*, where tomatoes showed repression of *PR1* and *PR4*, suggesting that infection with pathogen would reduce the plant self-defense mechanism, hence promoting the development of the disease.

In this study, when the interaction of *R. pseudostellariae* with *T. harzianum* ZC51 was analyzed, *PR10*, *PRSTH-21*, *PR1a, PR4*, *CH4*, and *CH5b* were up-regulated. Others have shown that *T. harzianum* T39 reduces the incidence of downy mildew of grapes by directly regulating the expression of defense-related genes (Perazzolli et al., 2011). Similarly, a study has reported the increase in the expression level of several defense-related genes in olive trees only when *T. harzianum* (Ths97) was applied together with the root rot pathogen *F. solani* (Amira et al., 2017). Other studies have shown that *Trichoderma* spp. may also trigger ISR in plants, mainly related to the expression of pathogenesis-related proteins (i.e., *PR1*, *PR2*, and *PR5*) (Hermosa et al., 2012; Mathys et al., 2012). Phenylalanine ammonia lyase (*PAL*) is one of the most widely studied enzymes involved in the process of plant disease resistance (Kim and Hwang, 2014). In this study, *T. harzianum* ZC51 did not change the expression of the *PAL* genes.

Compared with the “simple” two-partner systems (i.e., plant–pathogen or plant–antagonist), the complex three-way interactions involving *Trichoderma*, plant, and pathogen has received less attention, and this model can better simulate the natural interactions occurring in soil (Vinale et al., 2008). In our study, when *T. harzianum* ZC51 and *F. oxysporum* were applied together on *R. pseudostellariae*, we observed an upregulation of all the analyzed genes with the exception of PAL3. Similar results in the experiments to bean (*Phaseolus vulgaris* L.) infected with *R. solani* and/or *Trichoderma* were observed. The level of expression of defense-related genes (*CH5b*, *CH1*, *PR1*, *PR2*, *PR3*, and *PR4*) were up-regulated. Marra et al. (2006) studied the three-way interaction of *Trichoderma* with plant and fungal pathogens using proteomics methods, and the results show that antagonistic fungi will reduce the production of some defense proteins but will lead to the accumulation of others (i.e., PR proteins). This suggests that even in the presence of pathogens, several mechanisms are induced in *Trichoderma* that potentiates its ability to elicit plant defense responses (Mayo et al., 2016). Our results indicate that *Trichoderma* activate plants’ defense responses and so could be an optimized defense strategy against different plant stress, including plant pathogens and monocropping disease.

## Conclusion

To sum up, this study revealed that the continuous monocropping of *R. pseudostellariae* favored the growth of pathogenic *F. oxysporum* but decreased the antagonistic fungi (*Trichoderma* spp.), which resulted in poor yield of *R. pseudostellariae.* The exogenous application of *T. harzianum* ZC51 increased the expression levels of genes (*PR10*, *PRSTH-21*, *PR1a*, *PR4*, *CH4*, and *CH5b*) previously involved in plant defense, leading to enhanced defense response and improved growth of the host plant. These findings can be useful to develop locally customized and innovative approaches to address major threats facing medicinal plant cultivation.

## Data Availability Statement

The raw data supporting the conclusions of this article will be made available by the authors, without undue reservation.

## Author Contributions

WL, JC, and SL conceived the study. JC, LZ, and ID wrote the manuscript. JC, QL, and JW performed the experiments. TW, LW, LZ, and HW performed the statistical analyses. XQ and YA were involved in field management and soil sampling. GP assisted in English correction. All the authors discussed the results and commented on the manuscript.

## Conflict of Interest

The authors declare that the research was conducted in the absence of any commercial or financial relationships that could be construed as a potential conflict of interest.
