# Supplementary material for: Antagonistic Activity of Trichoderma spp. Against Fusarium oxysporum in Rhizosphere of Radix pseudostellariae Triggers the Expression of Host Defense Genes and Improves Its Growth Under Long-Term Monoculture System
Source: Front Microbiol. 2021 Mar 15;12:579920. doi: 10.3389/fmicb.2021.579920 (PMC8005620; doi:10.3389/fmicb.2021.579920)
Supplement: Supplementary file 1 [file Data_Sheet_1.PDF]

Supplementary Material

Antagonistic activity of *Trichoderma* spp. against *Fusarium oxysporum* in rhizophere of *Radix pseudostellariae*, trigger the expression of host defense genes and improve its growth under long term monoculture system

Jun Chen[1, 2, 3], Liuting Zhou[1, 2,], Israr Ud Din[5], Yasir Arafat[1, 3, 4], Qian Li[1, 2, 3], Juanying Wang[1,2], Tingting Wu[1, 2, 7], Linkun Wu[1, 2, 3], Hongmiao Wu[1,2,3], Xianjin Qin[2,3], Ganga Raj Pokhrel[6], Sheng Lin[1,2]*, and Wenxiong Lin[1,2,3]*

[1]College of Life Sciences, Fujian Agriculture and Forestry University, Fuzhou 350002, Fujian, P. R. China.

[2]Fujian Provincial Key Laboratory of Agroecological Processing and Safety Monitoring, Fujian Agriculture and Forestry University, Fuzhou 350002, Fujian, P. R. China.

[3]Key laboratory of crop genetic breeding and comprehensive utilization of the ministry of education, Fujian Agriculture and Forestry University, Fuzhou 350002, Fujian, P. R. China.

[4]Key Laboratory of Crop Ecology and Molecular Physiology, Fujian Agriculture and Forestry University, Fuzhou 350002, Fujian, P. R. China.

[5]Institute of Biotechnology and Genetic Engineering (IBGE), the University of Agriculture Peshawar, Peshawar 25130, Pakistan.

[6]Tribhuvan University, Birendra Multiple Campus, Department of Chemistry, Chitwan, Nepal.

[7]Fujian Provincial Academy of Environmental Science, Fujian Provincial Technology Center of Emission Storage and Management, Fujian, P. R. China

*Correspondence:

Wenxiong Lin and Sheng Lin

Fujian Agriculture and Forestry University,

Email: lwx@fafu.edu.cn, linsh@fafu.edu.cn

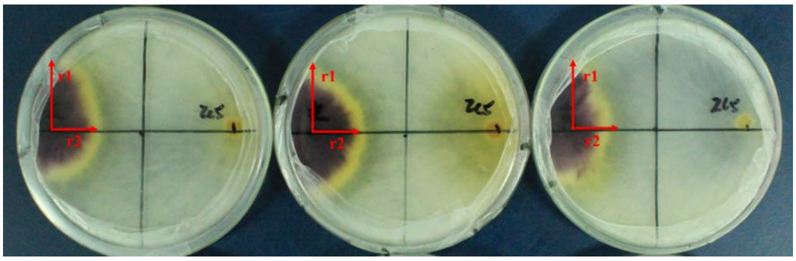

Supplementary Figure S1. Evaluation of antagonistic activity of *Trichoderma* (right) against *F. oxysporum* (left). The percentage of inhibition (PI) was calculated as % PI = [(r1−r2)/r1] x 100.

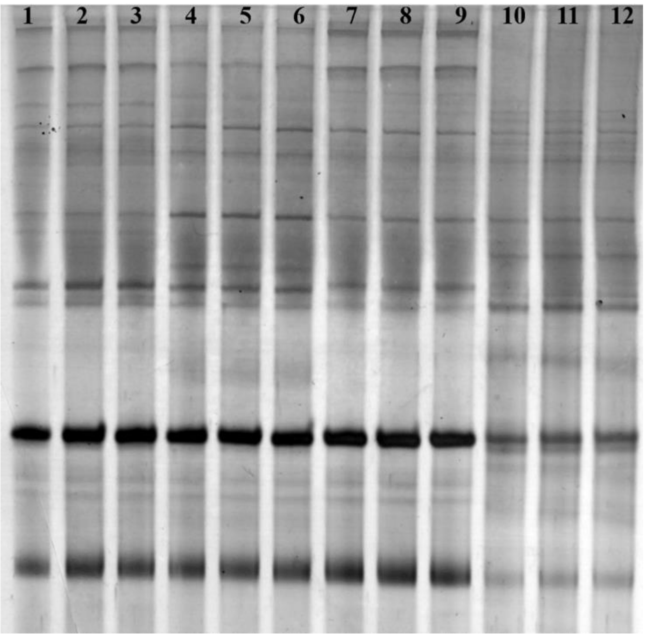

Supplementary Figure S2. *Trichoderma* community structure based on specific ITS denaturing gradient gel electrophoresis (DGGE). Lanes 1, 2 and 3 represent with no plantation of *R. pseudostellariae* (CK); lanes 4, 5 and 6 represent plot *R. pseudostellariae* cultivated in fresh soil (FP); lanes 7, 8 and 9 represent plot with *R. pseudostellariae*, monocultured for two consecutive years (SP); lanes 10, 11 and 12 represent plot with cultivation of *R. pseudostellariae* for three consecutive years (TP).

Supplementary Table S1 Taxon-specific primer sets and their thermal cycling conditions for quantitative PCR.

Primer	Sequence (5´- 3´)	Thermal conditions	Reference
Fusarium oxysporum			
ITS1-F	CTTGGTCATTTAGAGGAAGTAA	95℃ for 10min; 95℃ for 50 s, 60.4℃ for 1 min , 72℃ for 1 min, 40 cycles	Lievens et al., 2005
AFP308R	CGAATTAACGCGAGTCCCAA		
***Trichoderma* spp.**			
DG	GGTCTGAGAGGATGATCAGT	95℃ for 10min; 95℃ for 1 min, 55℃ for 1 min , 72℃ for 1 min, 40 cycles	Teng et al., 2015
DT	TTAGCTCCACCTCGCGGC		
tef1			
EF1	ATGGGTAAGGARGACAAGAC	95℃ for 3 min; 95℃ for 30 s, 53℃ for 30 s, 72℃ for 1 min, 35 cycles; 72℃ for 10 min	O'Donnell et al., 1998
EF2	GGARGTACCAGTSATCATGTT		
**ITS**			
ITS1F	CTTGGTCATTTAGAGGAAGTAA	95℃ for 3 min; 95℃ for 30 s, 55℃ for 30 s, 72℃ for 1 min, 35 cycles; 72℃ for 10 min	Gardes and Bruns, 1993; White et al., 1990;
ITS4	TCC TCC GCT TAT TGA TAT GC		
rpb2			
fRPB2-5f	GAYGAYMGWGATCAYTTYGG	95℃ for 3 min; 95℃ for 30 s, 58℃ for 30 s, 72℃ for 1 min, 35 cycles; 72℃ for 10 min	Liu et al., 1999
fRPB2-7cr	CCCATRGCTTGTYYRCCCAT		

References

Gardes, M., and Bruns, T.D. (1993). ITS primers with enhanced specificity for basidiomycetes-application to the identification of mycorrhizae and rusts. *Mol. Ecol.* 2: 113-118. DOI: 10.1111/j.1365-294x.1993.tb00005.x

Lievens, B., Brouwer, M., Vanachter, A. C., L évesque, C. A., Cammue, B. P., and Thomma, B. P. (2005). Quantitative assessment of phytopathogenic fungi in various substrates using a DNA macroarray. *Environ. Microbiol.* 7: 1698-1710. DOI: 10.1111/j.1462-2920.2005.00816.x

Liu, Y. J., Whelen, S., Hall, B. D. (1999). Phylogenetic relationships among ascomycetes: evidence from an RNA polymerse II subunit. *Mol. Biol. Evol.* 12: 1799. DOI: 10.1093/oxfordjournals.molbev.a026092

O'Donnell, K., Cigelnik, E., Nirenberg, H. I. (1998). Molecular systematics and phylogeography of the *Gibberella fujikuroi* species complex. *Mycologia.* 903: 465-493. DOI：10.2307/3761407

Teng, Y., Luo, Y., Ma, W., Zhu, L., Ren, W., Luo, Y., et al. (2015). *Trichoderma* reesei FS10-C enhances phytoremediation of Cd-contaminated soil by *Sedum plumbizincicola* and associated soil microbial activities. *Front Plant Sci.* 6: 438. DOI: 10.3389/fpls.2015.00438

White, T.J., Bruns, T., Lee, S., and Taylor, J. (1990). Amplification and direct sequencing of fungal ribosomal RNA genes for phylogenetics. PCR protocols: a guide to methods and applications. 1, 315-322.

Supplementary Table S2 Primers designed for Real-Time PCR analysis.

Gene	Function name	Primer	Primer sequence	References
actin	actin	Actin-F	CTGTATTTACGCTCAGGTGG	Qin et al., 2017
		Actin-R	CATTGTGCTCAGTGGTGG	
PAL1	phenylalanine ammonia-lyase 1	PAL1-F	CAACCACTCATCAGACCCAC	This study
		PAL1-R	GAGCCACTGTCAATGTTTCC	
PAL3	phenylalanine ammonia-lyase 3	PAL3-F	ATGTTGGAAGCCATCACC	This study
		PAL3-R	AGCCCAGCAATGTAGGAG	
CH5	chitinase 5-like	CH5-F	CCCAATCCAACTAACCTG	This study
		CH5-R	ACTTGACACGGCTCCTAA	
CH4	Chitinase class IV	CH4-F	CTCTAGTTACCCTAGTTTCG	This study
		CH4-R	TCAGTAGCCAAGATTTCC	
CH1	Chitinase 1	CH1-F	TTCTTATGCTCCTAATCACCC	This study
		CH1-R	CGAGTAGTCCCACAGTATCCA	
PR10	pathogenesis-related protein 10	PR10-F	AGGTGATGTACTCCGAGGCG	This study
		PR10-R	GCAACGAGGTATTCAGAAGCAG	
PRSTH-21	pathogenesis-related protein STH-21	PRS-F	GTGGAGTTACCCCAGCAAG	This study
		PRS-R	CAGAGTCAACGTCACCCTCA	
PR1a	pathogenesis-related protein 1a	PR1a-F	AACAATACGCAAACGAAAG	This study
		PR1a-R	AACCAAGACGAACCGAGT	
PR4	pathogenesis-related protein PR-4	PR4-F	TATGCCTAGTTGTCGGTGGT	This study
		PR4-R	GTTGTTTGTGCTCCTGTTCC	
